# Optimization of protein isolation by proteomic qualification from *Cutaneotrichosporon oleaginosus*

**DOI:** 10.1007/s00216-019-02254-7

**Published:** 2019-12-04

**Authors:** Dania Awad, Thomas Brueck

**Affiliations:** Werner Siemens-Lehrstuhl für Synthetische Biotechnologie, Technische Universität München, Lichtenbergstrasse 4, 85748 Garching, Germany

**Keywords:** Proteomics, *C. oleaginosus*, Oleaginous, Yeast, Method optimization, Protein purification

## Abstract

In the last decades, microbial oils have been extensively investigated as a renewable platform for biofuel and oleochemical production. Offering a potent alternative to plant-based oils, oleaginous microorganisms have been the target of ongoing metabolic engineering aimed at increasing growth and lipid yields, in addition to specialty fatty acids. Discovery proteomics is an attractive tool for elucidating lipogenesis and identifying metabolic bottlenecks, feedback regulation, and competing biosynthetic pathways. One prominent microbial oil producer is *Cutaneotrichosporon oleaginosus*, due to its broad feedstock catabolism and high lipid yield. However, this yeast has a recalcitrant cell wall and high cell lipid content, which complicates efficient and unbiased protein extraction for downstream proteomic analysis. Optimization efforts of protein sample preparation from *C. oleaginosus* in the present study encompasses the comparison of 8 lysis methods, 13 extraction buffers, and 17 purification methods with respect to protein abundance, proteome coverage, applicability, and physiochemical properties (pI, MW, hydrophobicity in addition to COG, and GO analysis). The optimized protocol presented in this work entails a one-step extraction method utilizing an optimal lysis method (liquid homogenization), which is augmented with a superior extraction buffer (50 mM Tris, 8/2 M Urea/Thiourea, and 1% C7BzO), followed by either of 2 advantageous purification methods (hexane/ethanol or TCA/acetone), depending on subsequent applications and target studies. This work presents a significant step forward towards implementation of efficient *C. oleaginosus* proteome mining for the identification of potential targets for genetic optimization of this yeast to improve lipogenesis and production of specialty lipids.

**Electronic supplementary material:**

The online version of this article (10.1007/s00216-019-02254-7) contains supplementary material, which is available to authorized users.

**Figure:**
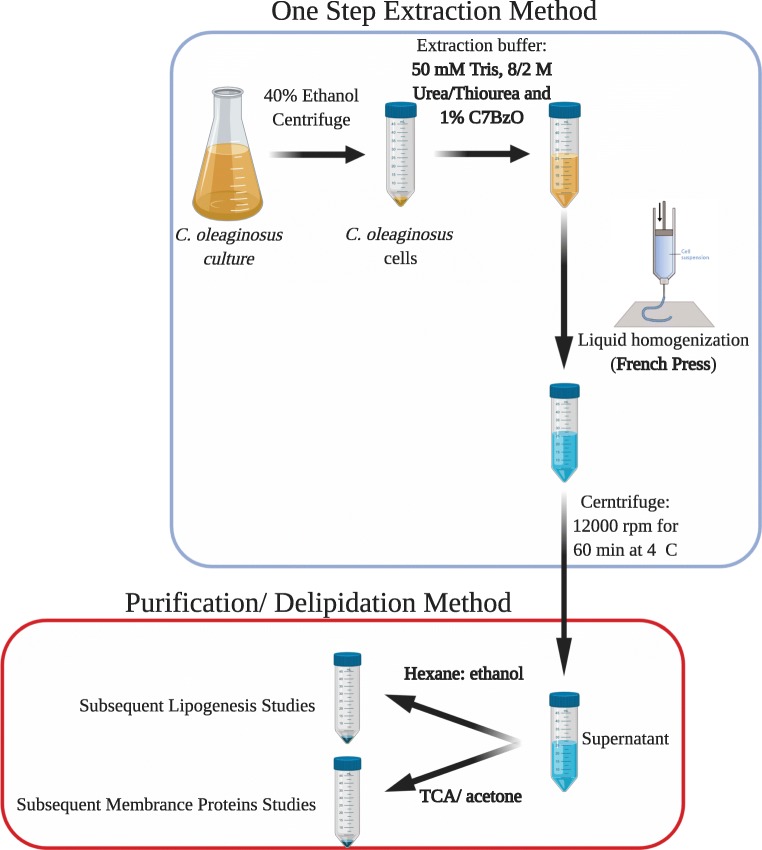
Graphical abstract

## Introduction

Climate change drives the development of sustainable bioprocesses in the chemical and pharmaceutical industry. To that end, microbial oils have been identified as a renewable alternative to petroleum-based chemical entities [1]. Leading strains in development are *Cryptococcus sp*., *Lipomyces sp.*, *Rhodotorula sp.*, *Rhodosporidium sp.* and *Trichosporon sp.* [2–4], and *Cutaneotrichosporon oleaginosus* (ATCC 20509) [5]. The latter yeast species, *C. oleaginosus* is metabolically capable of converting a wide range of carbohydrates (glucose, galactose, cellobiose, xylose, sucrose, and lactose) and complex biomass-derived residual substrates (whey, glycerol, volatile fatty acids, ethanol, and N-acetylglucosamine) into lipids (60% w/w)[1, 6, 7]. The fatty acid profile of *C. oleaginosus* mimics that of plant-oils with 16–33% C16:0 and 43–57% C18:1 and therefore can be used to generate biodiesel and oleochemical specialty products [8].

Development of an industrially relevant strain requires a comprehensive understanding of the complex genomic, proteomic, and metabolic system networks that determine and control microbial oleaginicity [3]. Finding novel genes and pathways committed to oleaginicity should facilitate strain engineering for improved lipid titers, robustness, and techno-economics of the microbial production of fatty acid derivatives [3]. Synergistically, the study of oleaginous yeast proteome is an increasingly attractive method for dissecting the molecular basis of lipogenesis [9]. For discovery proteomics, sample preparation procedures should comprehensively and reproducibly capture the protein repertoire with minimal artifactual modification, degradation, and contamination [10].

Most oleaginous yeast species possess robust cell walls composed mainly of polysaccharides (up to 90% w/w). These recalcitrant complex of sugar matrices maintain the mechanical strength, which conventionally require harsh physiochemical lysis systems. Hence, disruption methods must take into consideration the origin of the cells, physical strength of the cell wall, processing volume, and compatibility with downstream processes [11]. Several disruption methods have been developed including mechanical, chemical, and enzymatic treatments [12] . Mechanical methods entail harsh destruction of cell walls in a non-specific manner [11]. Industrial grinders include Waring® Blender and Playtron Mixer [13, 14]. On a smaller scale, pulverizing cells in liquid nitrogen, liquid homogenization, and ultrasonication has shown prospect for yeast cells [9, 11]. Alternatively, physical disruption of bonds within cell walls can be achieved by high temperatures and/or repeated cycles of freezing and thawing [12]. In a more specific manner, enzymatic digestion is a gentle method of disintegration [15, 16]. Nevertheless, the high cost and restricted availability of enzymes limit their utilization in large-scale processes [11]. Furthermore, these enzymes are commonly unfavorable, especially in discovery proteomics, due to complications arising from interference with downstream processes [17]. In addition to enhanced permeability, chemical treatments offer an increased value to protein extraction–protein solubilization [18]. Simultaneous cell lysis and protein solubilization is thus achieved by combining physical and chemical (detergents) methods [11]. Unfortunately, reconstituting membrane proteins into these solvent systems has been shown to be a tedious and challenging task. This is reflected in the under-representation of structural and mechanistic records for membrane proteins in Protein Data Bank (less than 1%) [18]. Given that severe solubility problems hamper the analysis of membrane proteins, successful discovery proteomics is thus reliant on careful selection of solubilizing detergents [19, 20].

As new detergents are continually being developed, choosing an appropriate detergent for discovery proteomic studies is hampered by the vast selection of detergents available today. Unfortunately, there is no ideal detergent for all applications and results often vary for the same application. The efficacy of detergents in extracting and solubilizing proteins can be further refined by additives such as chaotropes (urea and thiourea) [20]. Trial and error is the best strategy to find the optimal extraction buffer and the use of a mixture of detergents should also be considered.

The high concentrations of detergents (0.5–4%) and chaotropes (5–8 M), typically required for efficient solubilization of elusive proteins, are incompatible with downstream processes, as they inhibit trypsin activity, suppress LC-ESI-MS ionization, compromise chromatographic separation, and generate high-abundance ions that interfere with MS analysis [21]. Hence, their elimination is as crucial for subsequent analytical manipulations as the removal of inherent interfering compounds (lipids, nucleic acids, phenolic compounds, carbohydrates, proteolytic and oxidative enzymes, and pigments). To minimize protein modifications and proteolysis arising from these compounds, purification method should also be optimized [22]. In addition to typical challenges of protein sample preparation arising from inherent protein heterogenecity, structural complexity, and instability, the high lipid content of *C. oleaginosus* presents an added challenge. Specifically, lipids and phospholipids have been associated with Matrix Effects (MEs). This phenomenon is commonly associated with Electrospray ionization (ESI) and is characterized by analyte signal suppression (false negative), enhancement (false positive), or mass deviation arising from matrix components. Accordingly, ME can dramatically influence the identification and quantification of proteins [23, 24]. Particularly as lipids co-precipitate with proteins, a high protein purity is difficult to achieve without extensive protocol optimization [9, 25].

Although purification of native proteins is a challenging exercise, several reliable approaches, such as salting out and precipitation by non-ionic hydrophilic polymers (PEG), have been established in protein biochemistry [26–28]. Purification by aqueous alcohols (methanol, ethanol, isopropyl alcohol, and butanol) and organic solvents such as acetone has been carried out for over a hundred years on commercial and industrial scale [22, 26, 29]. Trichloroacetic acid (TCA), nonetheless, is more effective at lower concentrations (15% for TCA, 75% for acetone and 90% for ethanol), yet requires a consequential step of removal by acetone [30]. The aggressive removal of non-protein compounds by TCA has been clearly demonstrated in diverse and complex biological samples such as soil [9, 31]. Protein extracts from various resistant plant tissues such as wood, olive leaves, maize, and hemp roots are also efficiently generated by phenol-based purification [9, 25]. Common protocols of lipid extraction have also been applied reversibly for delipidation of protein extracts [32]. Rigorous testing demonstrated that replacing chloroform with methyl-tert-butylether (MTBE), which is non-toxic and non-carcinogenic, delivers similar or better recovery of lipids from human blood and brain samples [33–35]. Further attempts aimed at reducing dangers of chloroform toxicity adopt hexane as a lipid carrier. The efficiency of this method has been demonstrated in the recovery of several lipid classes from leaf tissues of Arabidopsis, tomato, soybean, and sunflower cake [36–38]. Despite the availability of diverse purification and delipidation methods for attaining adequate protein quality, finding the optimal method is a laborious comparative task, as different methods may result in depletion of particular protein species and relative enrichment of others [39].

In spite of the increased number of studies aimed at understanding lipogenesis in various oleaginous yeast species, scarce records for the optimization of protein purification and delipidation methods are deposited in literature. Thick floating lipid pads are often observed during the extraction of proteins from highly oleaginous yeasts. Nonetheless, partial loss of hydrophobic and membrane proteins is thus assumed when this lipid pad is scooped out and discarded prior to purification [40]. Proteomic studies of *Rodosporidium toruloides*, *Yarrowia lipolytica*, and *Mucor circinelloides* adopt common purification systems, which are optimized for reluctant plant tissues such as TCA/acetone and biphasic chloroform/methanol method without prior method optimization [2, 3, 41–44].

As it is difficult to predict which protocol could result in optimal proteome coverage of the non-model oleaginous yeast *C. oleaginosus*, we provide a comprehensive study that qualifies protein preparation methods and their downstream applicability based on qualitative and quantitative methods of proteins and lipids. This study addresses the three most challenging aspects of protein sample preparation by examining 7 methods of disintegration methods, 13 extraction buffers for protein solubilization, and 17 methods of purification/delipidation for optimal protein sample preparation from the oleaginous yeast *C. oleaginosus.*

## Materials and methods

### Yeast strain and cultivation media

*Cutaneotrichosporon oleaginosus* ATCC 20509 (from the culture collection of Werner Siemens Chair of Synthetic Biotechnology–WSSB, TU, Munich) was maintained on YPD (yeast extract peptone dextrose) agar plates (20 g L^−1^ peptone, 20 g L^−1^ agar, 20 g L^−1^ glucose, 10 g L^−1^ yeast extract). A single colony was initially cultured in 125 mL Erlenmeyer flask holding 50 mL YPD liquid medium at 28 °C and in a rotary incubator at 120 rpm for 24 h. Lipid accumulation was induced by subsequent inoculation in 125 mL Erlenmeyer flask holding 50 mL of Minimal-Nitrogen Media MNM (40 g L^−1^ glucose, 0.75 g L^−1^ yeast extract, 1.5 g L^−1^ MgSO_4_·7H_2_O, 0.4 g L^−1^ KH_2_PO_4_, 0.22 g L^−1^ CaCl_2_·2H_2_O, and trace elements 1.2 mg L^−1^ (NH_4_)_2_SO_4_, 0.55 μg L^−1^ ZnSO_4_·7H_2_O, 24.2 μg L^−1^ MnCl_2_·4H_2_O, 25 μg L^−1^ CuSO_4_·5H_2_O) prepared according to [1].With a starting optical density of 0.1, measured at 600 nm, cultivation was sustained for 96 h at 28 °C in a rotary incubator at 120 rpm.

### Experimental design

#### Sample preparation

Yeast cells from 10 mL cultures were pelleted and washed twice with 40% ethanol. Processing conditions included the addition of a volume of 3:1 extraction buffer: pellet of the buffer (25 mM Tris-HCl, 5 mM 2-Mercaptoethanol, 5 mM EDTA, and 100 μM PMSF, pH 8.0) for all samples subjected to the varied attempted lysis methods. To ensure minimal proteolysis and protein modification, operating conditions were restricted to low temperatures. Optimization of protein extraction was portrayed at two levels: lysis method and extraction buffer.

#### Disintegration methods

Cell lysis was attempted in 8 different methods including (1) thermolysis at 121 °C for 15 min using autoclave (Systec VE-150, Germany), with an estimated processing time of 80 min, (2) 7 cycles of freezing at − 20 °C and thawing at 4 °C, and (3) lyophilization using Alpha 2-4 LD plus Lyophilizer (Martin Christ, Germany) following sample freezing at − 80 °C. Mechanical disruption covered (4) liquid homogenization via EmulsiFlex-B15 French Press (Avestin, Canada) with 4 consecutive passes at 8 bar, (5) pulverizing with Mortar and Pestle in liquid nitrogen, (6, 7) sonication of iced-bathed samples using (Bandelin Sonopuls, Germany) for 5 intermittent cycles of 30 s acoustic waves delivered at 90% power (in the presence and absence of glass beads, probe height 1 cm from container base), and (8) 5 expedited autolysis by intermittent cycles of 30 s vortexing in the presence of glass beads.

The homogenates were then centrifuged at 12,000 rpm for 60 min at 4 °C and supernatants were precipitated by TCA/acetone according to [45]. The efficiency of cell lysis was monitored by 4 parameters: visualizing pelleted cell debris under light microscopy and attending to their granularity, measuring the concentration of extracted soluble proteins and assessing their quality by SDS-PAGE.

#### Extraction buffer

Following the determination of the optimal lysis method, identical sample handling proceeded for the optimization of extraction buffer. A list of all examined detergents and their general properties is presented in Table 1. Briefly, ionic detergent (2% SDS), anionic detergents (1% Triton X-100, 2% Tween® 20, and 2% Tween® 80), and zwitterionic detergents (CHAPS, 1% C 7BzO, and 1% SB3-10) were compared for their extraction and solubilization potential. Molar equivalence of the aforementioned detergents amounting to 2% w/w was also compared, labeled here as “2% Mixture.” The concentrations of detergents used in this study were selected based on manufacture recommendation; Triton X-100, Tween® 20, and CHAPS (Carl Roth, Germany); SDS (SERVA, Germany); and Tween® 80 (AppliChem, Germany). Additionally, the solubilization prospective of urea was assessed single-handedly and concurrently with thiourea (Carl Roth, Germany). The combined effect of detergents and chaotropic agents was also considered with a mixture of 2% SDS and 8 M urea. SB3-10 and C7BzO were examined as constituent of Extraction Reagents Type 2 (ERT2) and 4 (ERT4), respectively (Sigma Aldrich, USA). Furthermore, ERT4 was freshly reproduced in the lab with and without 50 mM Tris-HCl. Restriction of protein oxidation and proteolysis was attained by addition of 5 mM 2-Mercaptoethanol and 100 μM PMSF to all compared buffers. All reagents used in this study are detergent-grade.

**Table 1:** Detergents examined in this study with their structural categories, properties, and target applications

Detergents	Structural category	Properties	Target
SDS	Ionic	Charged head group	Protein–protein interactions
Tween 20	Non-ionic	Uncharged head group	Lipid–lipid and lipid–protein interactions
Tween 80			
Triton X-100			
CHAPS	Zwitterionic	Net neutral charge head group	Combined properties of ionic and non-ionic
C7BzO			
SB3-10			

The efficiency of extraction buffer to solubilize whole proteome was assessed by measuring the concentration of protein extracts by Bradford assay and visual inspection of corresponding bands on SDS-PAGE, following TCA/acetone precipitation according to [45]. Additional evaluation criteria accounted for the count of spectra, unique peptides, and proteins following in-solution tryptic digestion and mass spectrometry.

#### Purification protocols

Protein extracts of equal volumes and concentrations, prepared via found optimal disintegration method and extraction buffer, were subjected to 17 purification methods. These methods, in addition to any deviations from adopted protocols are recorded in Table 2. All reagents used are of high purity/HPLC quality when applicable. The quantity and quality of recovered proteins was attended to by Bradford quantitation and SDS-PAGE. Furthermore, purification methods were compared based on the number of identified proteins following in-solution digestion and LC MS/MS analysis, GRAVY scores, pI, and MW distributions of identified proteins, in addition to Clusters of orthologous groups (COGs) and Gene Ontology (GO) analyses. The extent of delipidation of each method was also assessed by measuring the FAME content in purified dry protein sample.

**Table 2:** Protein purification methods attempted in this study

Purification method	Protocol modifications^c^	Source
Ethanol	9 volumes of ethanol	[46]
100% acetone	–	[47]
80% acetone	–	[48]
TCA/acetone	13.3% TCA	[45]
Optimized TCA/acetone	Glass beads	[10]
Phenol:methanol:ammonium acetate	–	[49]
Chloroform:methanol	–	[50]
PCI^a^	Collect organic phase	[51]
Methanol:MTBE:water	–	[35]
Butanol/di-isopropyl ether	Collect aqueous phase	[52]
Sequential^b^	–	[9]
PEG 6000	–	[27]
Hexane:ethanol	Ethanol replaced isopropanol, 60 °C incubation for 30 min	[38]
Size exclusion	Modified PES 10 kDa, 500 μL	VWR, Germany
Ammonium sulfate	–	[53]
DISSOLVAN® 7:ethanol	Adapted protocol from (38)	Clariant
DISSOLVAN® 5:ethanol	Adapted protocol from (38)	Clariant

^a^*PCI* phenol/chloroform:isoamyl alcohol

^b^Sequential (TCA/acetone + phenol)

^c^Modifications made to adopted protocols

### Analytical methods

#### Microscopy

The extent of cell ruptures during optimization of lysis method was evaluated by visualizing pelleted cell debris under oil immersion light microscope (Motic, China) equipped with Moticam 5.0 MP. The thickness of *C. oleaginosus* cell wall was investigated using a JSM-7500F scanning electron microscope (SEM) (JEOL, Japan). SEM was equipped with an accelerating voltage of 1, 2, or 5 kV and a secondary detector.

#### Flow cytometry

Lysis efficiency was estimated based on granularity of cell fragments following lysis methods described above. Initially, lysates were passed through a 40 μm nylon mesh prior analysis on S3 Cell Sorter (Bio-Rad, USA), equipped with 488 nm/100 mW laser beam. Automated alignment verification and drop delay determination was carried out using ProLine™ Universal Calibration Beads (Bio-Rad, USA). A drop delay of 33.16 was found to provide optimal flow with an event rate of 60,000. Forward-scattered light (FSC) and side-scattered light (SSC) trigger threshold was set to 0.05 with a voltage of 284 W for the former and 294 W for the latter. Sheath fluid (2.978 g L^−1^ disodium EDTA, 2.069 g L^−1^ potassium phosphate, 2.266 g L^−1^ Potassium chloride, 18.852 g L^−1^ sodium phosphate, and 64.985 g L^−1^ sodium chloride) carried 100 μL of each of 8 generated lysates, in addition to a sample of intact *C. oleaginosus* cells, through the cytometer. FSC AND SSC were acquired on a log_10_ scale using ProsSort^TM^ software (Version 1.5). The region occupied by intact pool of *C. oleaginosus* cells dictated the quadrant reserved for cells, which withstood disintegration forces, and served as basis in the calculation of lysis efficiency.

#### Lipid quantitation

The extent of lipid contamination in protein pellets, following various purification methods, was measured by accounting for the sum of fatty acid methyl esters (FAMEs), obtained by methanol transesterification. The transesterification protocol was originally adopted from [46]. and modified in our lab by [47]. FAME profiles were analyzed on a GC-2025 gas chromatograph from Shimadzu (Nakagyo-ku, Kyōto, Japan) with flame ionization detector. One microliter sample was applied by AOC-20i auto injector (Shimadzu) onto a ZB-WAX column (30 m, 0.32 mm ID; 0.25 μm df; phenomenex (Torrance, CA, USA)). The initial column temperature was 150 °C (maintained for 1 min). A temperature gradient was applied from 150–240 °C (5 °C min^−1^), followed by 6 min maintenance at 240 °C. Fatty acids were identified according to retention times of the authentic standard: Marine Oil FAME Mix (Restek, USA). Individual FAME concentrations were based on peak areas relative to Methyl Nonadecaanoate C19 (Sigma, Germany), which was incorporated as an internal standard in all samples. Percent lipid was calculated from the sum of individually identified FAMEs with respect to pellet dry weight.

#### Protein quantification and SDS-PAGE

Protein concentrations were quantified using Bradford protein assay (Carl Roth, Germany) following TCA/acetone precipitation. Bovine serum albumin (BSA) was chosen for modeling standard curves and measurements were recorded in triplicates in 96-well plates on EnSpire® Multimode Plate Reader (PerkinElmer, USA). Protein extracts were conveyed on 12% one-dimensional SDS polyacrylamide gel electrophoresis, using Bio-Rad Mini-Protean II Equipment and PageRuler ™ Unstained Protein Ladder (ThermoFischer Scientific, USA), to assess the gross qualitative variances in protein profiles. After electrophoresis, gels were stained with Coomassie brilliant blue (CBB) R-250.

#### Shotgun proteomics

In-solution tryptic digestion was carried out on 1 mg of purified protein pellet resolved in 50 mM ammonium bicarbonate. Digestion proceeded with Sequencing Grade Modified Trypsin (Promega, USA) at a ratio of 1:20 trypsin:protein w/w at 37 °C overnight following sequential reduction and alkylation in 10 mM DTT (95 °C for 5 min then 60 °C for 30 min) and 20 mM iodoacetamide (at room temperature in the dark for 20 min). Termination of tryptic treatment was assumed by incubation on ice for 5 min. Peptides were then vacuum dried and reconstituted in 1% formic acid. Trypsin and other contaminants were eliminated from peptides by centrifugal filtration using low protein binding, modified PES centrifugal filters with 10 kDa cutoff (VWR, USA).

LC MS/MS was performed on filtrates using an Ultimate 3000 RSLCnano system (Dionex/Thermofischer) coupled online to LTQ Orbitrap XL mass spectrometer. Tryptic digests of 100 ng were loaded onto Acclaim^TM^ PepMap^TM^ trap column (100 C18; 3 μm, 75 μm × 20 mm) at a flow rate of 5 μL min^−1^ prior to reverse-phase separation on Acclaim^TM^ PepMap^TM^ column (100 C18; 2 μm, 75 μm × 500 mm) at a flow rate of 200 nl min^−1^. Reverse-phased buffer system combined 0.1% trifluoroacetic acid aqueous solution (buffer A) and acetonitrile with 0.1% trifluoroacetic acid (buffer B). A separation cycle of 150 min gradient (0–4% buffer B for 7 min, 4–35% buffer B for 102 min, 35–65% buffer B for 3 min, 65–90% buffer B for 2 min; after maintaining buffer B at 90% for 10 min, the entire system was then re-equilibrated by 4% buffer B for 26 min) and an inter-sample 60 min blank delivered peptides to the Nanospray Flex Ion Source. MS parameters allowed a scan range of 350–1400 Da with resolution of 60,000. The mass spectrometer was set such that one MS scan was followed by 6 MS/MS scan events; opt for most intense ion signal. MS/MS parameters limited minimum signal intensity to 1000, isolation width to 2 Da and allowed for dynamic exclusion.

### Bioinformatics

Raw MS/MS files were searched in Proteome Discoverer 2.2 software (Thermo Fisher Scientific, Germany) against *C. oleaginosus* database, downloaded from UniProt (https://www.uniprot.org/proteomes/, 8317 proteins) using SEQUEST. Search parameters allowed 10 ppm and 0.02 Da tolerance for the precursor and fragment, respectively. Semi-cleavage was tolerated up to 2 missed cleavages for trypsin with cysteine residues (57.0215 Da) and methionine residues (+ 15.9949 Da) as constant and variable modifications, respectively. Cross correction values (Xcorr) of at least 1.2 (+ 1), 1.9 (+ 2), 2.3 (+ 3), and 2.6 (> + 4), Δ*C*_n_ cutoff value of 0.05 and high confidence peptide filters with a minimum length of 6 amino acids were applied to ensure less than 1% peptide level FDR. The hydrophobicity of proteins was based on the Grand Average of Hydropathy (GRAVY) scores, whereby protein sequences from MS/MS data of identified proteins were imported into GRAVY web-based tool (http://www.gravy-calculator.de). Clusters of orthologous groups (COGs) were created with the aid of WebMGA, a web-based tool for fast metagenomic analysis [48]. The biological processes and molecular functions of identified proteins for Gene Ontology (GO) analysis were assigned using WEGO with baker’s yeast as reference strain, following blast against *C. oleaginosus* database, interpro, mapping, and annotation in Blast2Go [49, 50].

## Results and discussion

### Disintegration methods

#### Lysis efficiency

**Fig. 1:**
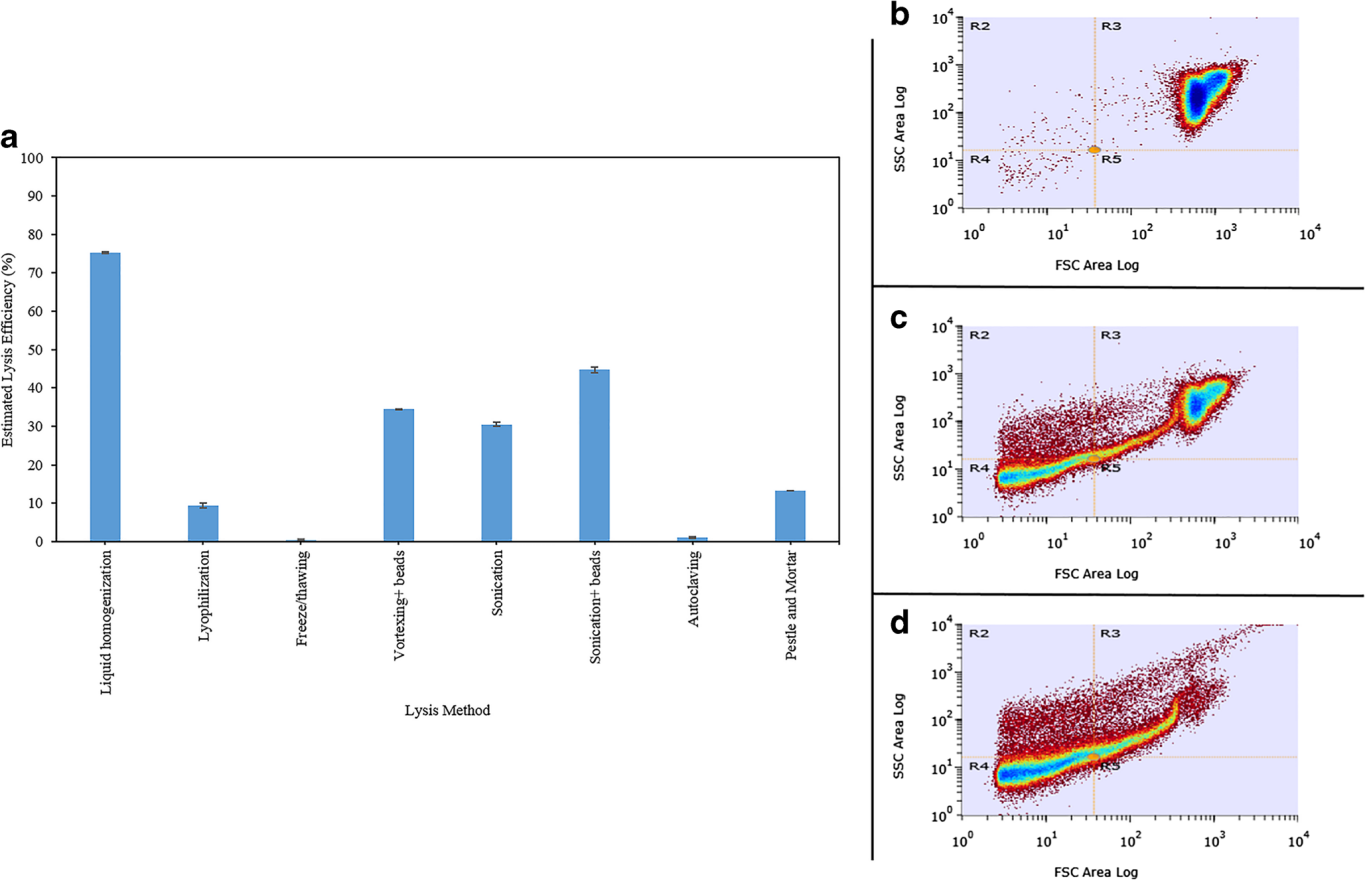
Estimated lysis efficiency plot (**a**) based on cell granularity measurements for untreated cells (**b**) and cells broken by sonication (**c**) and liquid homogenization (French Press) (**d**). Calculations are based on forward and secondary scatter plot division into 4 quadrants with quadrant (R3) reserved for unbroken cells. Scatter plots of remaining lysis methods are available in Fig. S2 of Supplementary Material

The extent of cell lysis has direct consequences on the overall quality of protein isolation process and is approached as the first bottleneck in proteomic analysis of cell wall–enclosed species [11]. The highest degree of fragmentation in this study was achieved by liquid homogenization with 75.2% efficiency, based on cell granularity measurements (Fig. 1a, d). Mechanical disruption is generally regarded as random and non-uniformal, yet it delivers maximal destructive impact applicable for many yeast and plant species to impair their robust cell walls [11]. In fact, the overall SDS-PAGE band intensities associated with French press disruption is exceptionally distinguishable from remaining methods that resulted in incomplete protein liberation (see Electronic Supplementary Material (ESM) Fig. S1b). Physical impairment and release of cellular components of yeast cells has been predominantly aided by glass beads [51]. In this study, coupling of sonication with glass beads generated lysis efficiency of 44.7%, compared to 30.5% for standalone sonication (Fig. 1a). The level of proteins released in this method amounts to 7 μg μL^−1^ and their validation by SDS-PAGE revealed faint band pattern inferior only to liquid homogenization (ESM Fig. S1). This method of disintegration is hence recommended in absence of costly homogenization instruments. However, temperature control is crucial and obligatory as most of the ultrasound energy, absorbed by the suspension, is translated to heat [11].

Manual grinding of crop in liquid nitrogen is regarded as the conventional disruptive method to combat the tensile strength of cellulose, chitin, and other polysaccharides constituting the plant cell wall [9]. This method of disintegration has been adopted protein sample preparation from *R. toruloides* for comparative proteomics [2]. With mere 13.3% overthrow success, minimal concentration of soluble proteins (1.8 μg μL^−1^), and scant protein representation on SDS-PAGE, this method is considered incompetent in accessing *C. oleaginosus* (Fig. 1 and ESM Fig. S1). Lyophilization, freeze/thawing cycles, vortexing in presence of beads, sonication, and thermolysis also failed to achieve their purpose in this analysis (Fig. 1 and ESM Fig. S1). The unreasonably high protein concentrations measured for samples that were subjected to lyophilization and thermolysis can be attributed to protein profile shifts through induction of heat and cold shock proteins (ESM Fig. S1a) [52]. Furthermore, formation of odorant compounds in reaction to thermal damage has been correlated with significant increase in the respective precursor amino acids leucine (3-methylbutanal), ornithine, and proline (2-acetyl-1- pyrroline) (Münch & Schieberle, 1998).

While lytic enzymes infer a more gentle and effective disintegration alternative, they were eliminated from this study as they impose additional downstream processing in proteomic exploration (removal of lytic enzymes and/or preparation of laborious MS exclusion lists) [53, 54]. Notably, pelleting of *C. oleaginosus*, for media elimination and washing, necessitated the use of 40% v/v ethanol to avoid loss of “cellular floaters” that result from high lipid content. This step of sample preparation bears no destructive impact on this yeast. Uncoupling the optimization of disintegration method from solubilization method, allowed for objective evaluation of lysis method independently from extraction buffer. The development of an efficient lysis method tailored for *C. oleaginosus* is also applicable to recover the yeast oil from cellular debris.

#### Cell wall thickness

**Fig. 2:**
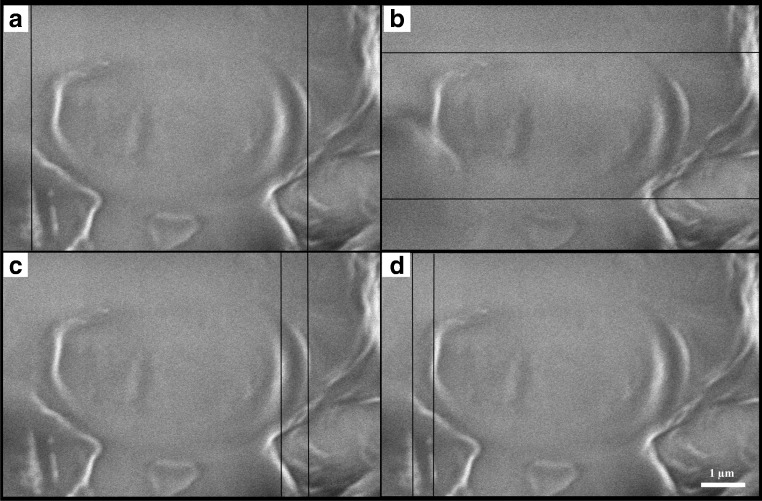
Morphology of *C. oleagin*osus visualized by a scanning electron microscope (SEM). Applied energy, 1.00 kV, LEI; detector, SEM/LM. Vertical and horizontal rulers mark cell borders in estimating cell diameter (**a**, **b**) and cell wall thickness (**c**, **d**). Scale bar = 1 μm at 14,000 x magnification

The strength of this yeast cell wall is undoubtedly demonstrated in ESM Fig. S3 with numerous “ghosts” visible under direct microscopy for attempted lysis methods. This is indicative of incomplete cell wall destruction and intracellular components retention. This prompted the measuring of this yeast cell wall thickness. *C. oleaginosus* cells are assumed to be elliptical spheroids with an average diameter ranging between 3.5 and 6.3 μm (Fig. 2a, b). The thickness of this yeast cell wall is threefold greater than the laboratory strain *S. cerevisiae* BY4741, measuring at 0.5–0.6 μm by scanning electron microscopy (Fig. 2c, d) [55]. Further studies are requisite for understanding the specific composition, mechanical properties, and molecular forces behind the strength of *C. oleaginosus* cell wall.

### Extraction buffer

In order to accent the degree of cell lysis and efficiently solubilize this oily yeast proteome, the use of detergents in extraction buffer is necessary. These two vital roles of detergents cannot be individually investigated, but their pooled outcome is marked by the refined quantity and quality of extracted proteins. While assessment of the former involves quantifying the concentration of protein extracts, the latter requires further downstream processes, including SDS-PAGE, in-solution digestion, and mass spectrometry. One-step extraction in lysis buffer was adopted in this study as it is considered the simplest and most useful straightforward procedure [10].

**Fig. 3:**
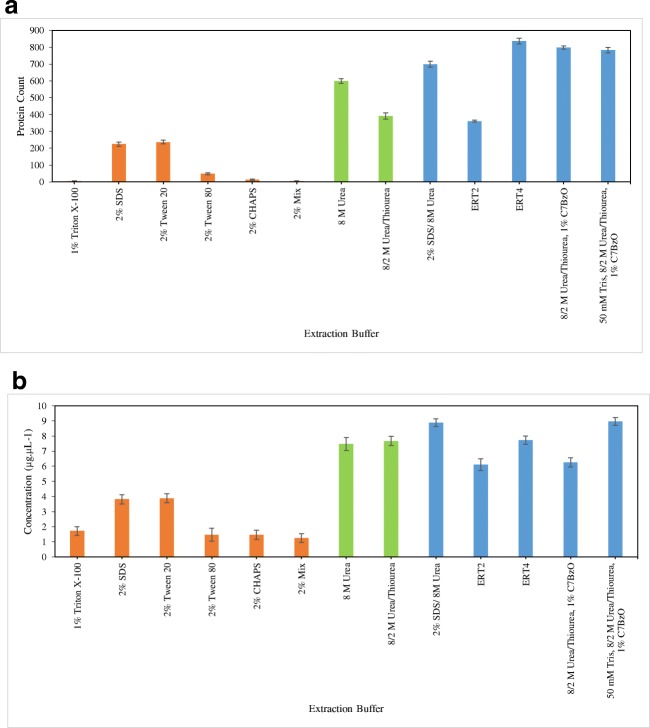
The count of total uniquely identified proteins (**a**) and the quantitation of whole proteome (**b**) extracted from *C. oleaginosus* with examined detergents. Calculations are based on Bradford assay per BSA standards curve (*R*^2^ value of 0.9914)

In contrast to the reported inefficiency of standalone chaotropes for handling complex membrane samples [20], urea extracted double the amount of proteins, in comparison with standalone detergents, with a concentration of 7.5 μg μL^−1^(Fig. 3b). Moreover, recorded increase in the number of protein bands for urea on SDS-PAGE (ESM Fig. S4e and f) implies the extraction of additional proteins. This was confirmed by LC MS/MS with the identification of 599 unique proteins (Fig. 3a). However, samples containing urea should not be exposed to temperatures higher than 37 °C as urea establishes equilibrium with cyanate in solution, which covalently modifies amino acid side chains in a reaction that is greatly accelerated by heat and alkaline conditions [56]. Thiourea does not appear to enhance the extraction potential of the buffer as it resulted with the extraction of 392 unique proteins only (Fig. 2a). The use of a chaotropic agents in the extraction of *C. oleaginosus* proteins has shown to be crucial with a 2.3-fold increase in protein concentrations resulting from coupling of urea with SDS (8.87 μg μL^−1^), compared to SDS treatment solely (3.81 μg μL^−1^) (Fig. 3b). This was concurrent with identification 699 and 224 unique proteins, respectively (Fig. 3a). Albeit the proven competence of this buffer, we recommend against it when downstream processes such as 2D-PAGE are in order, on the account of irreversible aggregation and precipitation of proteins brought about by SDS removal [19].

Unlike ionic and non-ionic detergents, zwitterionic detergents have been demonstrated to have additional value in downstream processes. Henceforth, the combined power of chaotropic agents and zwitterionic detergents, SB3-10 and C7BzO, was attended to by evaluating two extraction buffers offered by Sigma Aldrich with claims of increasing efficiency, ERT2 and ERT4, respectively. ERT4 performed superiorly on LC MS/MS with 839 identified proteins, compared to 360 proteins for ERT2 (Fig. 3b). C7BzO, the detergent constituent of ERT4, is named amongst best candidates for protein solubilization [20]. Its extraction and solubilization power and compatibility with IEF and 2D electrophoresis make it a promising detergent for proteomic analysis of *C. oleaginous* [57]. Further evaluations of freshly prepared in-house replica of ERT4 were aimed towards cost reduction of the extraction protocol. Accordingly, the significance of Tris at 50 mM in the extraction buffer was upheld with 2.7 μg μL^−1^ increase in protein concentration and the identification of 16 additional unique proteins (Fig. 3). In the light of the presented results, subsequent optimization of protein sample preparation from *C. oleaginosus* proceeded in a one-step extraction method via liquid homogenization in an extraction buffer containing 50 mM Tris, 8/2 M Urea/Thiourea, and 1% C7BzO.

### Purification method

#### Purification efficiency

Protein purification, often achieved by precipitation, is the final and fairly demanding step in protein isolation. This work compares the purification efficiency of 17 methods in (1) the elimination of contaminants and secondary metabolites, namely, lipids for this oleaginous strain, and (2) delivering a protein sample with highest protein coverage and minimal loss. In addition to widely applied purification methods including sequential purification, our optimization efforts comprise methods commonly used for lipid, DNA, and RNA extractions—modified to retain protein fractions and efficiently eliminate the otherwise collected fraction. These methods are listed in Table 2. It must be noted that interfering substances constitute one of the major problems in performing a Bradford assay [58]. For this reason, when purity of protein sample is in question, the intensity of band patterns of resolved proteins on SDS-PAGE provide more meaningful information on the quantity and quality of protein sample. For this reason, an SDS-PAGE is presented in Fig. 3c to evaluate the suitability of the purification methods for gel applications.

**Fig. 4:**
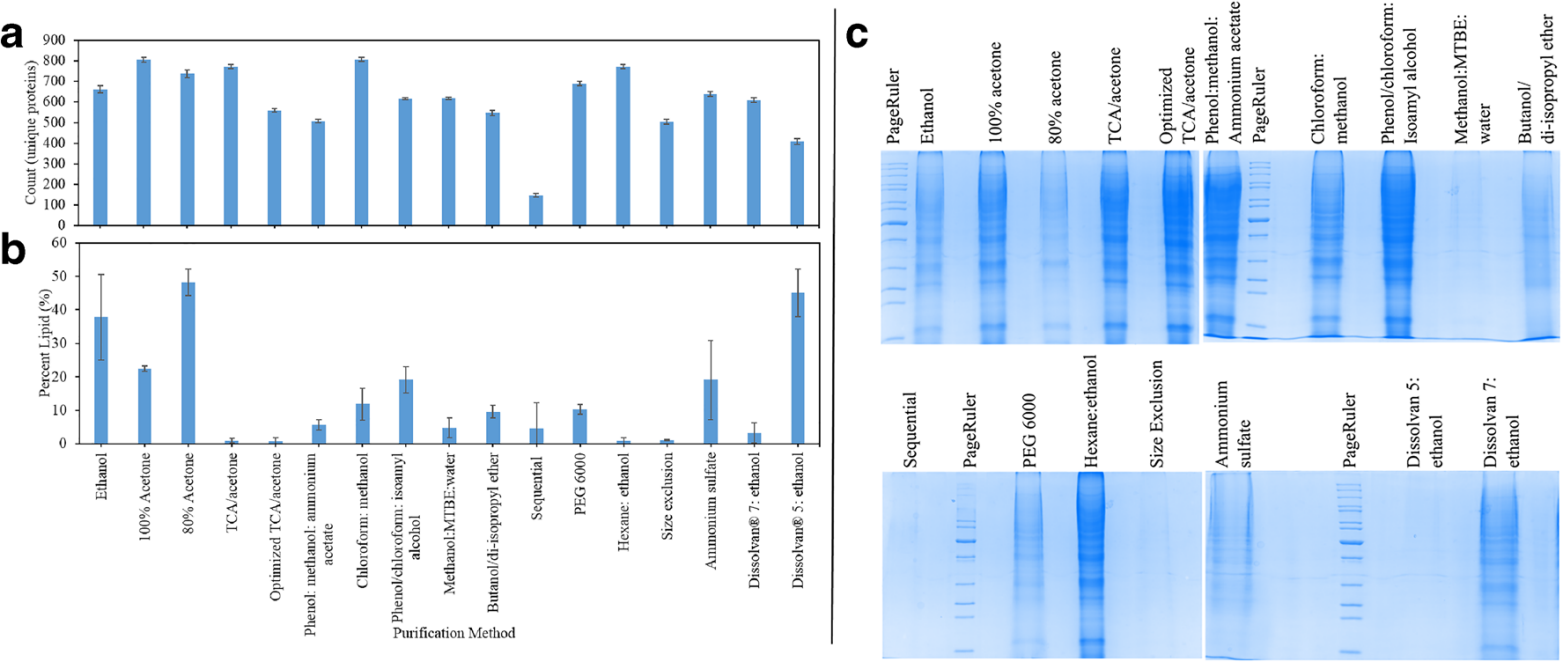
The count of total unique proteins identified following purifications (**a**). Lipid content (%) of purified protein samples, calculated by summation of FAMEs to biomass (g FAMEs/g dry yeast weight) (**b**)**.** Composite of 1D SDS-PAGE analyses for proteins purified by ethanol (a), 100% acetone (b), 80% acetone (c), TCA/acetone (d), optimized TCA/acetone (e), phenol:methanol:ammonium acetate (f), chloroform:methanol (g), phenol/chloroform:isoamyl alcohol (h), methanol:MTBE:water (i), butanol/di-isopropyl ether (j), sequential (k) PEG 6000 (l), hexane:ethanol (m), size exclusion (n), ammonium sulfate (o), Dissolvan 7:ethanol (p), and Dissolvan 5:ethanol (q), whereby L represents the protein ladder (**c**)

Precipitation by 100% acetone resulted in one of the highest number of identified unique proteins (805 unique proteins) (Fig. 4a). However, acetone tends to co-precipitate different types of lipids with proteins [59]. This is clearly depicted in Fig. 4b with 20–50% (g FAMEs/g dry protein pellet weight) of precipitate content detected as lipids for acetone precipitations. As lipids are highly associated with ME, which severely compromises the analysis performance, their elimination is at the core of method development and validation [23]. Precipitation by 80% acetone was aimed at simplifying the final re-dissolution step; however, this method does not achieve improved results, neither in protein identification (737 unique proteins) nor in delipidation (48.1% (g FAMEs/g dry protein pellet weight)). Similarly, ethanol precipitation, best known in fractionating human serum, delivered intermediate results with 662 uniquely identified proteins and fade SDS-PAGE bands (Fig. 4a, c) [26]. Resembling acetone precipitation, high lipid content (37.9% g FAMEs/g dry yeast weight) was detected in the precipitate of ethanol precipitation (Fig. 4b).

With the advantage of reduced solvent volume, TCA/acetone resulted in the second highest record of identification with 770 unique proteins (Fig. 4a). In comparison with other organic solvents and aqueous alcohols, this solvent system efficiently rids the protein precipitate from lipids with less than 1% of FAMEs (g FAMEs/g dry protein pellet weight) detected, as shown in Fig. 4b. However, this method irreversibly incorporates DNA, yielding DNA-protein aggregates, which cause precipitation, bad focusing, and protein streaking [59]. For this reason, when downstream processes include gel applications, this purification method is not recommended. In an attempt to overcome the laborious re-dissolution step, Hao et al. optimized TCA/acetone method by incorporating a subsequent step: the hard precipitate was ground into fine powder to enlarge the contact area between the precipitate and the washing solvent [10]. When applied to *C. oleagino*sus protein extract, this method delivered inferior results compared to standard TCA/acetone precipitation (Fig. 4) and protein loss was attributed to non-specific protein adsorption to glass beads.

Salting out by ammonium sulfate and PEG resulted with faint and highly distorted band patterns on SDS-PAGE; 637 and 688 identified unique proteins, respectively (Fig. 4a, c). One common drawback of these common methods is the difficulty of salt and PEG removal, with the former entailing laborious dialysis in a cold room against several volumes of buffer over long period of time and the latter requiring chromatographic steps on ion-exchange or affinity columns, ultrafiltration or salt-induced phase separation (Burgess 2009) [27]. Furthermore, the density of high concentrations of ammonium sulfate approaches that of protein aggregates resulting in floating of precipitate. Since this problem is further exacerbated in presence of high lipid content, this method should also be avoided when dealing with protein samples from oleaginous yeasts [26]. PEG 6000 was chosen for this study as larger polymers offer no advantage yet further increase the solution viscosity [27]. Purification by size exclusion using 10 kDa centrifugal filters, although gentle and straightforward, delivered subpar results with 505 identified unique proteins and undetected protein bands on SDS-PAGE (Fig. 4a, c). This method suffered from technical difficulties due to the presence of high lipid content as a lipid pad formed, blocking the filter.

Liquid-liquid extraction systems, aimed at DNA, RNA, and lipid isolation are highly exploited to efficiently eliminate these biomolecules in preparation of high-quality protein samples [21, 60]. These methods are appealing in preparation of protein samples from oleaginous yeasts. As such, the most common delipidation technique—chloroform:methanol—delivered one of the highest protein coverage (comparable with precipitation by 100% acetone) with 806 uniquely identified proteins and decent resolution of separated protein bands on SDS-PAGE (Fig. 4a, c). Nevertheless, this method is superior to precipitation by 100% acetone as it rids the protein sample from 88.2% (g FAMEs/g dry protein pellet weight) of the lipid content. In fact, this method has also been shown to improve the resolution of 2D-PAGE protein spots, when applied to low-density lipoproteins [61]. However, chloroform carcinogenicity poses considerable health risks for laboratory personnel [35]. Replacement of chloroform with MTBE promises more contact between solvents and biomolecules in a one-phase system [35]. However, MTBE is less efficient than the chloroform in the purification of the *C. oleaginosus* proteins. Similarly, the most common procedure used for delipidation of plasma, protein solutions, or cell culture medium makes use of butanol/di-isopropyl ether solvent system. Although this solvent system have been successfully employed in the defatting of whole buckwheat seeds, it has failed to improve protein yield of *C. oleaginosus* (546 unique proteins) and does not appear compatible with gel application (Fig. 4a, c) (Ma et al. 2006 ) [22]. Hexane/ethanol was reported to extract lipids with high efficiency from numerous plant species [37, 38]. This method delivered similar results as TCA/acetone in the number of uniquely identified proteins (770 proteins) and delipidation efficiency (less than 1% FAMEs (g FAMEs/g dry protein pellet weight)) (Fig. 4a, b). However, this method is more applicable than TCA/acetone in subsequent gel applications (Fig. 4c). High PSM counts were observed for hexane:ethanol (and ethanol) and these denote peptides identified repeatedly [62]. Demulsifier base chemistry technology was also attempted given its renowned application in petroleum and gas industry for separation of crude oil refining [63]. Furthermore, protein purification with the use of demulsifiers has been under-reported in literature [64]. Two demulsifying agents offered by Clariant, DISSOLVAN® 5 and 7, were compared in this study. These surfactants identified 407 and 608 unique proteins, respectively (Fig. 4a). While DISSOLVAN® 7 eliminated 96.7% (g FAMEs/g dry protein pellet weight) of lipids from protein sample, DISSOLVAN® 5 showed minimal delipidation efficiency, comparable with precipitation by ethanol and 80% acetone. Although protein purification by DISSOLVAN® 7 suffers from considerable protein loss, it can be attempted when preservation of protein conformation is required [64].

Phenol extraction methods, standalone or in combination with chloroform, have high cleanup capacity—especially for nucleic acids—given their original purpose. This is evident is the quality of SDS-PAGE protein bands delivered by phenol:methanol:ammonium acetate and phenol/chloroform:isoamyl alcohol purifications (Fig. 4c) [65]. However, the count of uniquely identified proteins for these methods (508 and 615, respectively) suggests that proteins were not properly retained (Fig. 4a). Phenol extraction also suffers from drawback of handling of toxic solvents (phenol and methanol) in addition to lengthy processing time (16 h). Lowest protein coverage in this study was associated with sequential purification, TCA/acetone followed by phenol:methanol:ammonium acetate. With the identification of 147 unique proteins and lack of SDS-PAGE protein representation, protein loss was owed to poor dissolution of the hard precipitate, resulting from TCA/acetone purification, in phenol (Fig. 4a, c).

#### High-performance purification methods

**Fig. 5:**
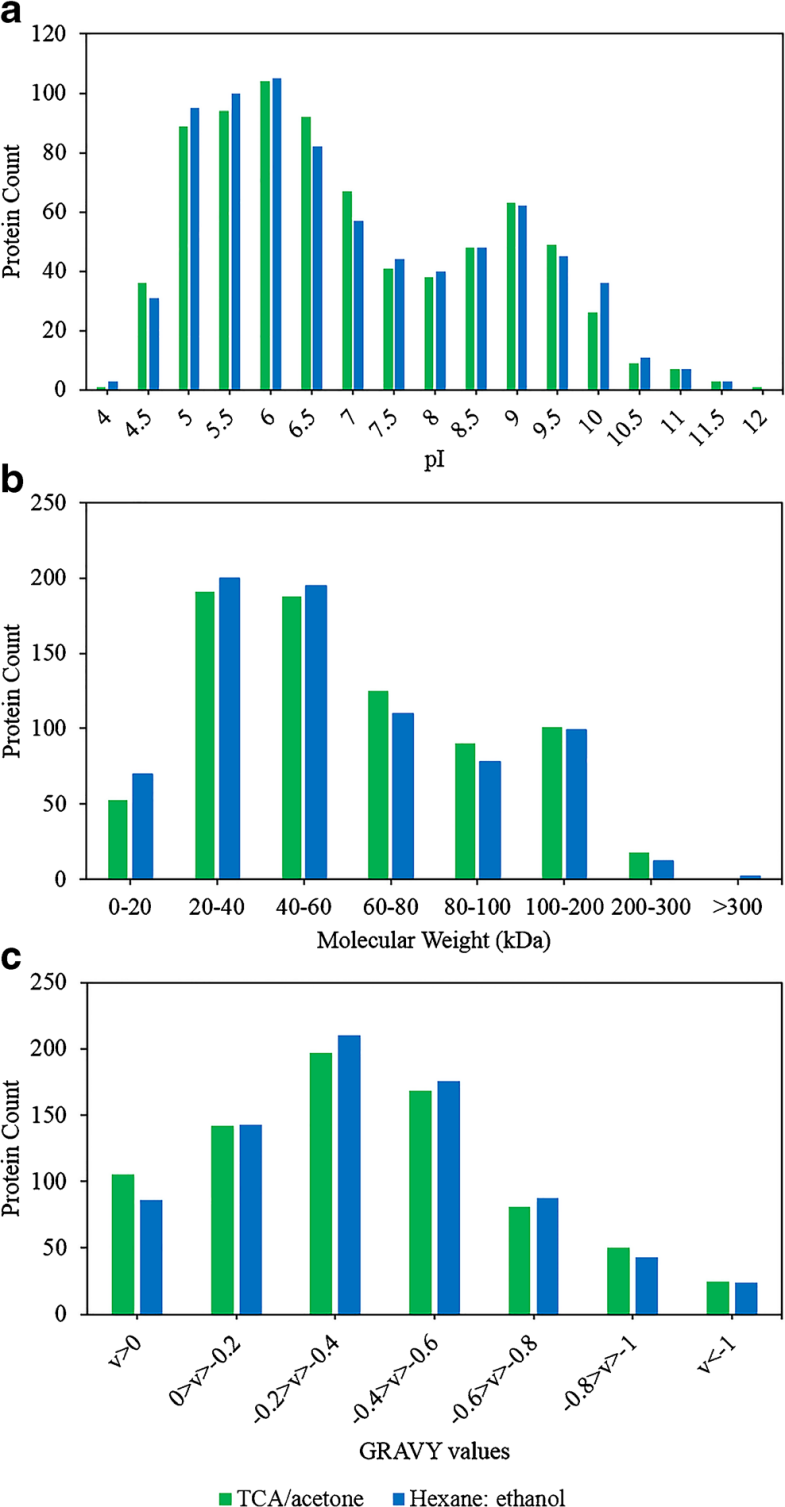
PI (**a**), molecular weight (**b**), and GRAVY score (**c**) distributions for whole *C. oleaginosus* proteome extracts obtained following purification by TCA/acetone and hexane:ethanol

Protein physiochemical heterogeneity is at the core of substantial differences in protein extracts amongst different methods [10]. For this reason, the hydropathicity, MW and pI distribution, Clusters of Orthologous Groups (COGs), and Gene Ontology (GO) analyses were performed to compare purification methods with highest protein coverage and delipidation efficiency—TCA/acetone and hexane:ethanol. These analyses did not reveal any clear patterns that might account for protein difference between methods, implying the complexity of protein extraction. As such, proteins purified by these methods equally fell in two pI ranges 4.5–6.5 and 8.5–10 (Fig. 5a). Yet, in accordance with literature, enrichment of basic proteins by this method in the pH range of 8.5–9.5 is detected [10]. The molecular weight of proteins detected for these methods fall in the range of 10–130 kDa, with proteins up to 300 kDa identified (Fig. 5b). Furthermore, GO analysis did not reveal any differences amongst these purification methods due to the limited choice of reference organisms (baker’s yeast) (ESM Fig. S5). Yet, this comparison can help make an informed choice of method based on the application and downstream processing. In that perspective, hydropathicity analysis revealed that TCA/acetone has retained the highest number of hydrophobic proteins (GRAVY > 0) (105 proteins) (Fig. 5a). Hence, this method is recommended when membrane proteins of *C. oleaginosus* are studied. Alternatively, COGs analysis performed in this study can help choose optimal method for targeted proteomic analysis of *C. oleaginosus* depending on number of identified genes associated with each method for the functional categories. Accordingly, hexane:ethanol method is recommended when energy production and conversion or carbohydrate transport and metabolism of *C. oleaginosus* are studied, as they achieved highest identification by this method, at 10.90 and 7.17%, respectively (ESM Fig. S6). Given that lipogenesis is triggered by nutrient starvation and represents a state energy preservation, this purification method is preferred when lipogenesis is target of proteomic studies. Furthermore, this method is also recommended for lipogenesis studies as the importance of the carbon source and concentrations for this yeast is upheld in nutritional starvation state [1]. Moreover, this method is more compatible with subsequent gel applications than TCA/acetone. This method is also recommended for proteomic studies of other oleaginous yeasts.

## Conclusion

This work comprises the first large-scale comparative study of extraction (8 lysis methods and 13 extraction buffers) and purification (17 methods) approaches of the non-model oleaginous yeast *C. oleaginosus*, with an emphasis on delipidation efficacy and method-specific differential proteomic profiles. The rigid cell wall (0.5–0.6 μm) of this yeast necessitated the use of liquid homogenization (French Press) to achieve a lysis efficiency of 75.2%. One-step extraction in lysis buffer was adopted to further augment the lysis efficiency. MS-based proteomic analysis revealed that Extraction Reagent Type 4 (50 mM Tris, 8/2 M Urea/Thiourea and 1% C7BzO) is superior in terms of proteome coverage, abundance, and subsequent gel application compatibility. MS qualification and delipidation efficiency of the large repertoire of purification methods revealed the superiority of TCA/acetone and hexane:ethanol. Further analysis pertaining to physiochemical heterogeneity revealed suitability of hexane:ethanol purification method for lipogenesis studies and TCA/acetone for membrane proteins. This paper marks the first attempt of method development for proteomic analysis of oleaginous yeasts, which is crucial for elucidating *de novo* lipogenesis and future genomic engineering aimed towards diverse applications in biofuel and oleochemicals.

## Electronic supplementary material

ESM 1 (PDF 1949 kb)
