# Supplementary material for: Optimization of protein isolation by proteomic qualification from Cutaneotrichosporon oleaginosus
Source: Anal Bioanal Chem. 2019 Dec 4;412(2):449–62. doi: 10.1007/s00216-019-02254-7 (PMC6992551; doi:10.1007/s00216-019-02254-7)
Supplement: Supplementary file 1 — (PDF 1949 kb) [file 216_2019_2254_MOESM1_ESM.pdf]

Analytical and Bioanalytical Chemistry

Electronic Supplementary Material

Optimization of protein isolation by proteomic qualification from *Cutaneotrichosporon oleaginosus*

Dania Awad, Thomas Brueck

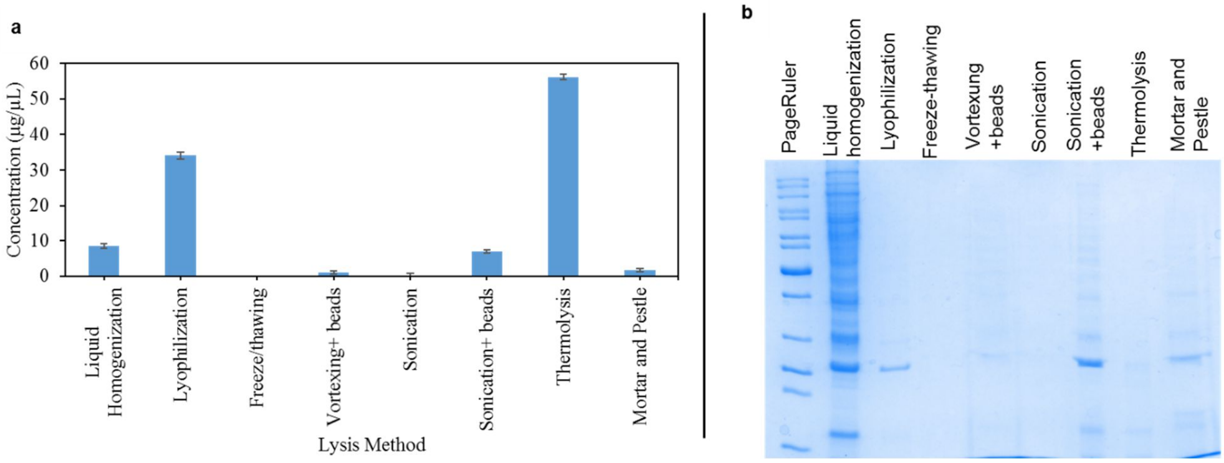

Fig. S1 Quantitation of soluble protein extracts from *C. oleaginosus* following attempted lysis methods (**a**). Calculations are based on Bradford assay per BSA standards curve (R^2 value of 0.9906). Composite of 1D SDS-PAGE analyses of soluble proteins (**b**) whereby L and a-h refer to protein ladder ,liquid homogenization (French Press), lyophilization, freeze/thaw, vortexing + beads, sonication, sonication + beads, thermolysis and mortar and pestle, respectively

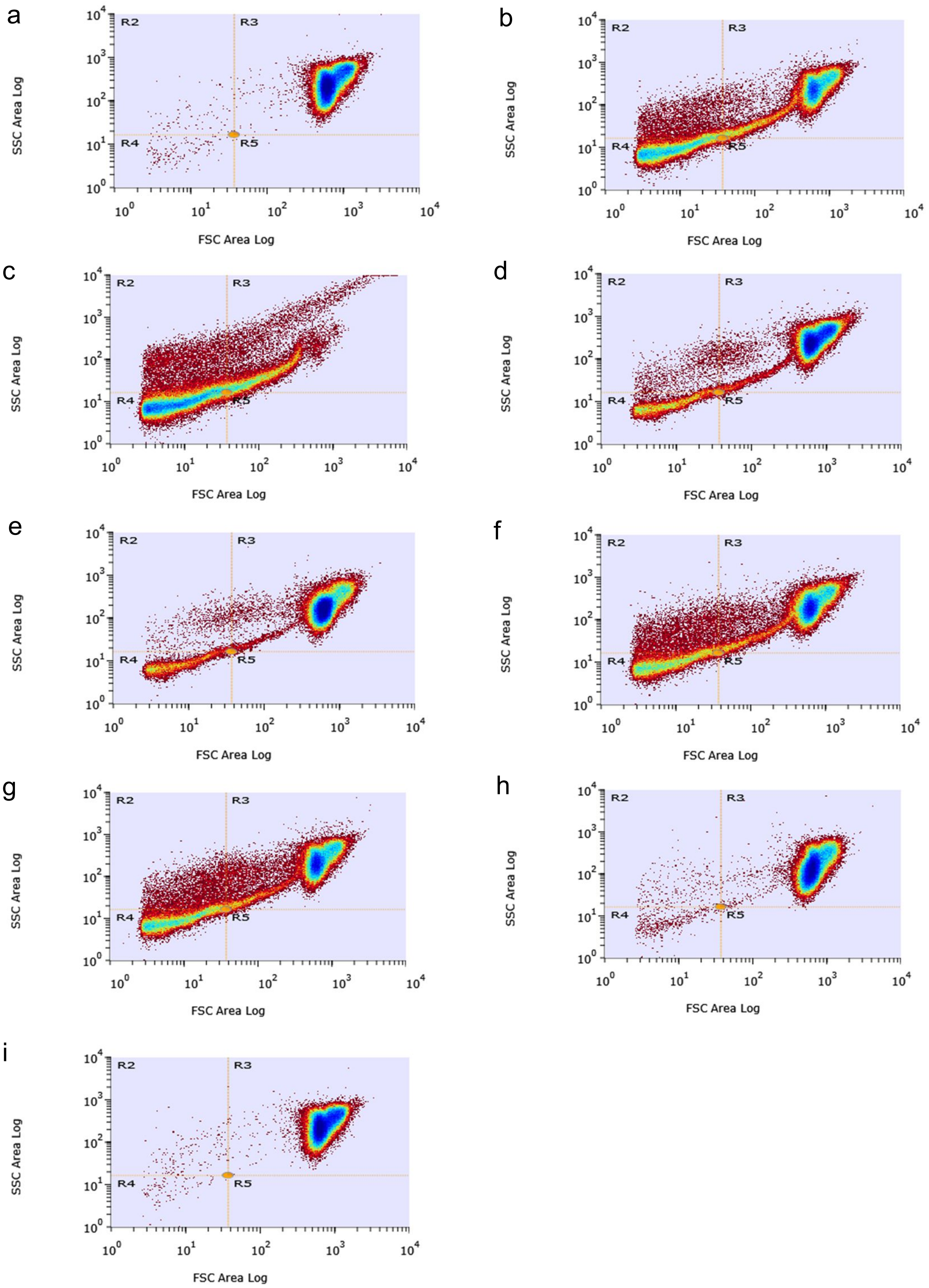

Fig. S2 Cell granularity based on forward and secondary scatter of *C. oleaginosus* prior lysis (**a**) and post lysis methods: (**b**) sonication, (**c**) French Press, (**d**) Mortar and Pestle, (**e**) lyophilization, (**f**) sonication + beads, (**g**) vortexing + beads, (**h**) thermolysis, (**i**) freeze/thaw

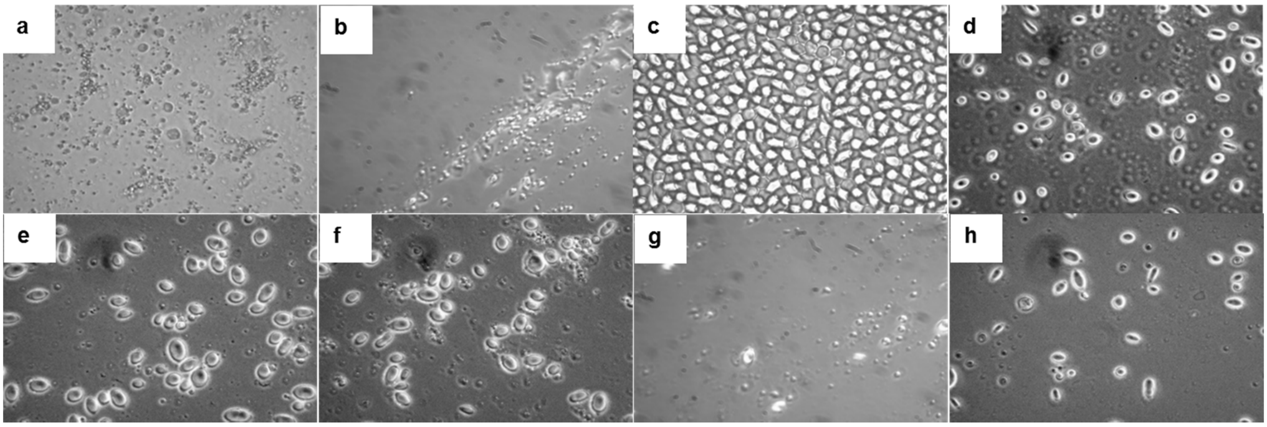

Fig. S3 Pelleted *C. oleaginosus* debris visualized by light microscopy under oil immersion (1000 times magnification) following attempted lysis methods: (**a**) French Press, (**b**) lyophilization, (**c**) freeze/thaw, (**d**) vortexing + beads, (**e**) sonication, (**f**) sonication + beads, (**g**) thermolysis, (**h**) mortar and pestle

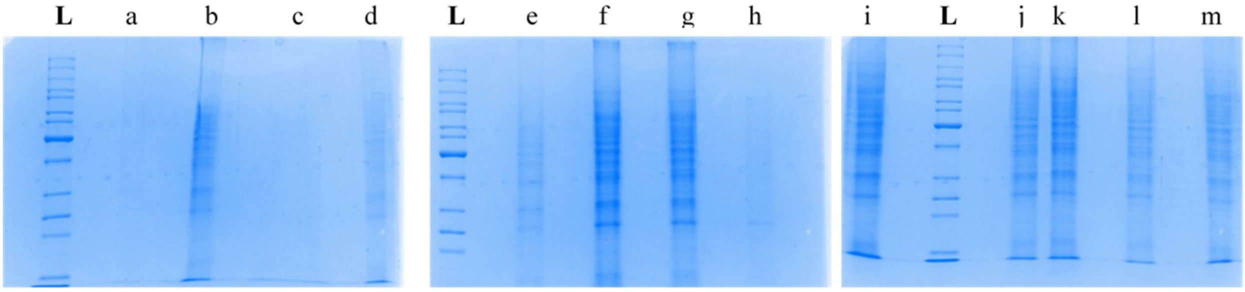

Fig. S4 Composite of 1D SDS-PAGE analyses (B) for proteins extracted by Triton X-100 (a), SDS (b), Tween® 80 (c), Tween® 20 (d), CHAPS (e), 8 M Urea (f), 8/2 M Urea/Thiourea (g), 2% Mixture (h), 2% SDS / 8 M Urea (i), ERT4 (j), 50 mM Tris, 8/2 M Urea/Thiourea, 1% C7BzO (k) and 8/2 M Urea/Thiourea, 1% C7BzO (l), ERT2 (m), whereby L represents PageRuler protein ladder

Table S1 An analysis of variance (one-way ANOVA) conducted in STATISTICA 7 for protein concentrations and MS qualification 1 measured following extraction by different detergents presented in Figure 3 and MS qualification 2 of different purification methods in Figure 4

**Dependent Variable (Protein Concentration)**	**Variance**
Multiple R	0.995727
Multiple R^2	0.991472
Adjusted R^2	0.987536
SS Model	7447.112
df Model	12
MS Model	620.5926
SS Residual	64.05325
df Residual	26
MS Residual	2.463587
F	251.9061
p	0.00
**Dependent Variable (MS qualification 1)**	**Variance**
Multiple R	0.999503
Multiple R^2	0.999007
Adjusted R^2	0.998549
SS Model	3796423
df Model	12
MS Model	316368.6
SS Residual	3772.667
df Residual	26
MS Residual	145.1026
F	2180.310
p	0.00
**Dependent Variable (MS qualification 2)**	**Variance**
Multiple R	0.998213
Multiple R^2	0.996429
Adjusted R^2	0.994748
SS Model	1311088
df Model	17
MS Model	81943.02
SS Residual	4699.333
df Residual	34
MS Residual	138.2157
F	592.8634
p	0.00

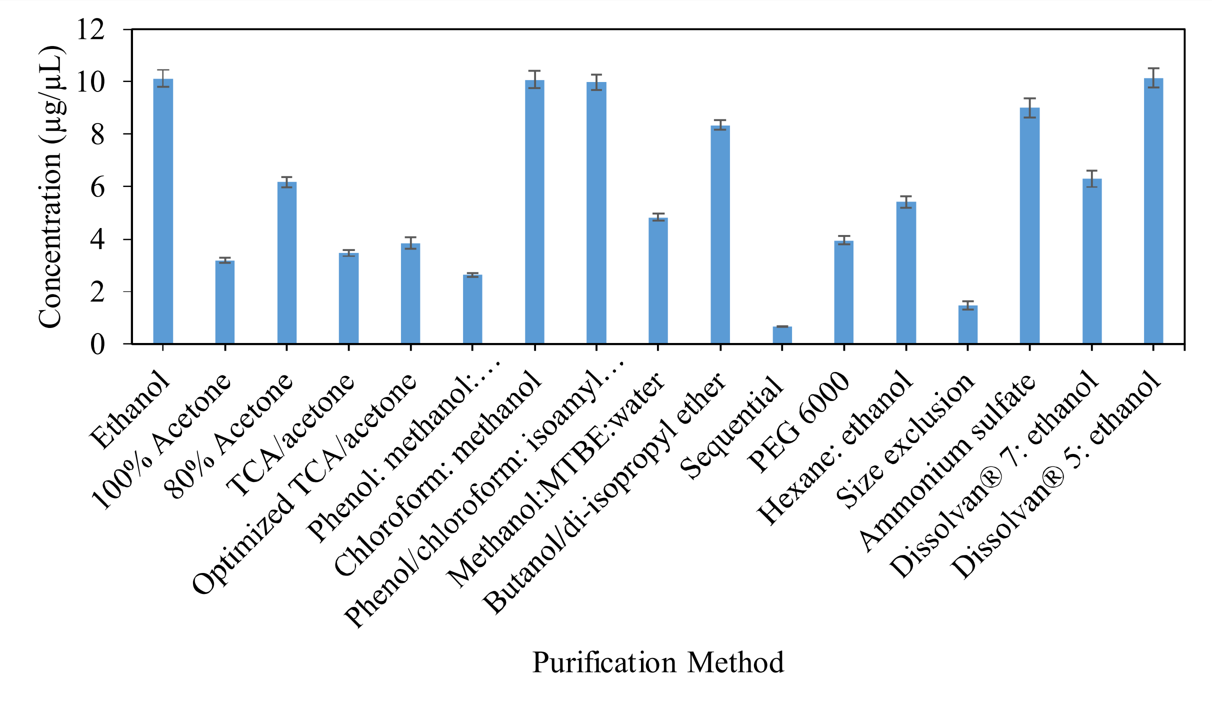

Fig. S5 Quantitation of whole proteome from *C. oleaginosus* following various purification methods. Calculations are based on Bradford assay per BSA standards curve (R^2 value of 0.9968)

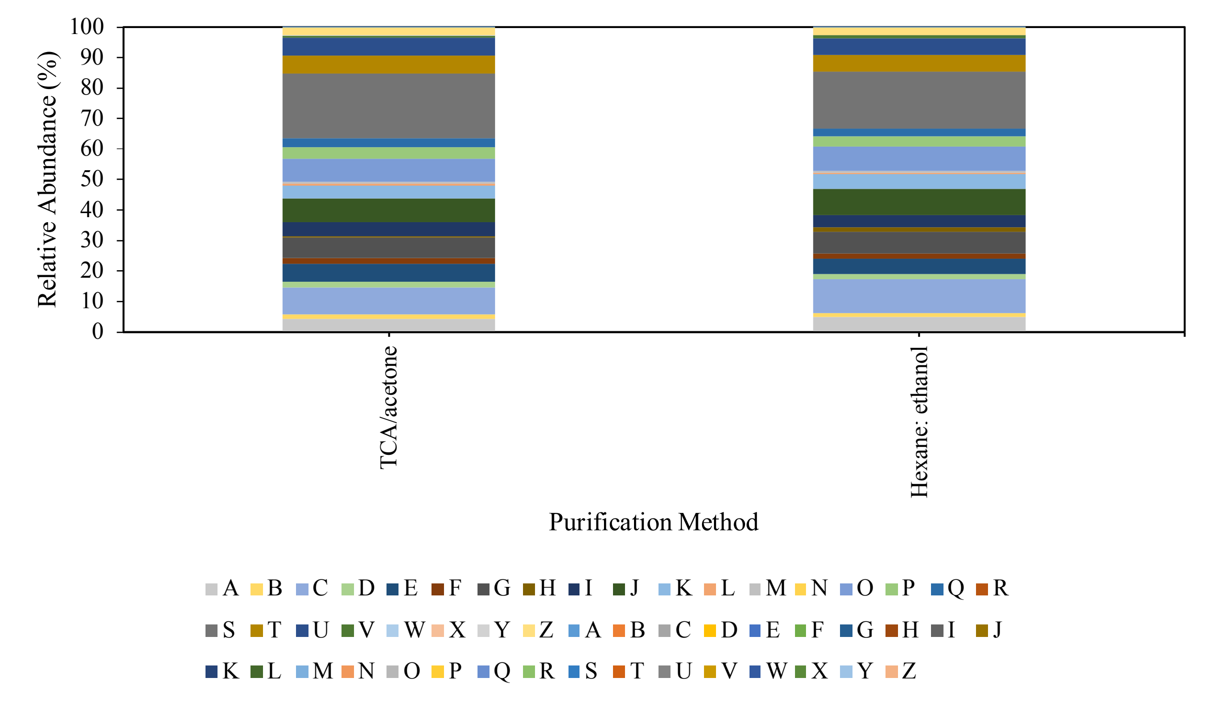

Fig. S6 COG Functional classification for whole *C. oleaginosus* proteome extracts obtained following purification by TCA/acetone and hexane: ethanol. One-letter abbreviations for the functional categories: A, RNA processing and modification; B, Chromatin Structure and dynamics; C, Energy production and conversion; D, Cell cycle control and mitosis; E, Amino Acid metabolism and transport; F, Nucleotide metabolism and transport; G, Carbohydrate

metabolism and transport; H, Coenzyme metabolism; I, Lipid metabolism; J, Translation; K, Transcription; L, Replication and repair; M, Cell wall/membrane/envelop biogenesis; N, Cell motility; O, Post-translational modification/protein turnover/chaperone functions; P, Inorganic ion transport and metabolism; Q, Secondary Structure; R, General Functional Prediction only; S, Function Unknown T, Signal Transduction; U, Intracellular trafficking and secretion; W, Extracellular structures; X, Mobilome/prophages/transposons [X] Y, Nuclear structure; Z, Cytoskeleton.

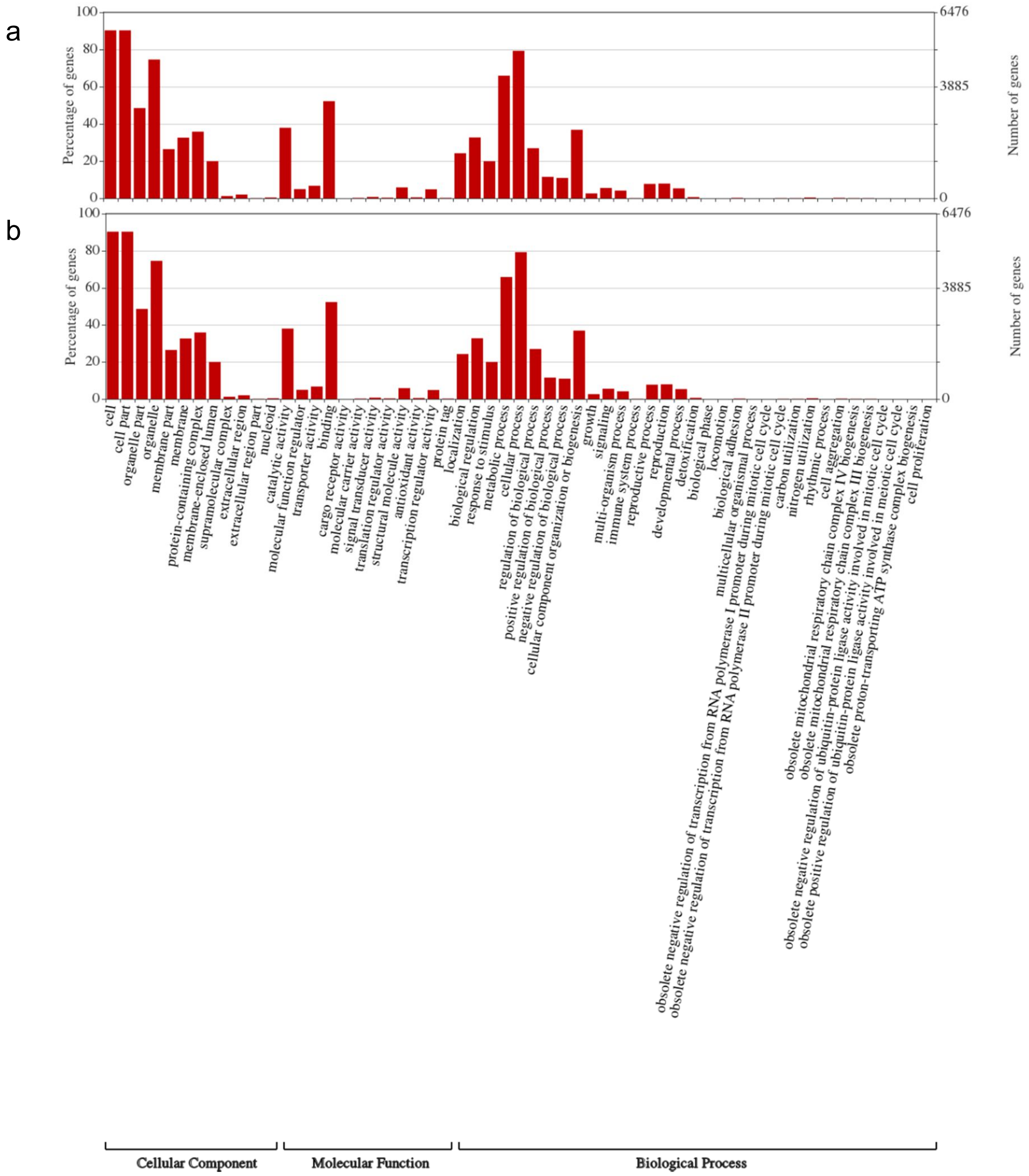

Fig. S7 Functional categorization of the genes matched to identified proteins purified by TCA/acetone (**a**) and hexane: ethanol (**b**). GO terms were assigned using BLAST2GO. The functional categorization was performed using WEGO (Web Gene Ontology Annotation Plot). Results are summarized in three main categories: cellular components, molecular functions and biological processes
